# Distribution of erythrocyte binding antigen 175 (*EBA-175*) gene dimorphic alleles in *Plasmodium falciparum* field isolates from Sudan

**DOI:** 10.1186/1471-2334-13-469

**Published:** 2013-10-09

**Authors:** Ahmed AM Adam, Ahmed AA Amine, Dina A Hassan, Waleed H Omer, Bakri Y Nour, Arulanantham Zechariah Jebakumar, Muntaser E Ibrahim, Nasreldin H Abdulhadi, Hiba S Mohamed

**Affiliations:** 1 Institute of Endemic Diseases, University of Khartoum, P.O. Box 102, Khartoum, Sudan; 2 Central Laboratory, Ministry of Science and Technology, P.O. Box 8076, Khartoum, Sudan; 3 University of Gezira, Wad Medani, Sudan; 4 Prince Sultan Military College of Health Sciences, Dhahran, KSA, Sudan; 5 College of Pharmacy, The National Rebat University, P.O. Box 55, Khartoum, Sudan

**Keywords:** Sudan, Erythrocyte binding antigen (EBA- 175), Dimorphic alleles (F and C), Glycophorin A (GPA)

## Abstract

**Background:**

The Erythrocyte Binding Antigen (EBA) 175 has been considered as one of the most important *Plasmodium falciparum* (*P. falciparum*) merozoite ligands that mediate invasion of the erythrocytes through their sialated receptor: Glycophorin A (GPA). The effect of the EBA 175 dimorphic alleles (F and C) on the severity of the disease is not yet fully understood. Therefore this study was designed to assess the distribution of the divergent dimorphic alleles of *P. falciparum* EBA-175 (F and C) in three different geographical areas in Sudan and the possible association of this dimorphism with the severity of the disease**.**

**Methods:**

A sum of 339 field isolates of *P. falciparum* obtained from patients in three different geographical areas in Sudan were screened for the dimorphic alleles (F, C) of the EBA-175 using nested PCR.

**Results:**

The percentage of F, C, and mixed F/C alleles were; 41%, 51%, and 8% respectively. F and C alleles showed significantly different distributions in the various geographic areas (*p = 0.00*). There was no significant association between malaria clinical manifestation and *P. falciparum* EBA-175 F and C alleles frequencies.

**Conclusions:**

This study showed a significant differential distribution of F and C alleles in different geographical malaria endemic areas. No significant association was observed between F and C alleles and different malaria phenotypes.

## Background

In the pathogenesis of the malaria parasite, *P. falciparum* merozoites invade the erythrocytes through multiple ligand-receptor interactions [1-4] providing an opportunity for using alternative invasion pathways if one has been blocked [5]. This illustrates the vital importance of using multiple ligand-receptor interactions for the *P. falciparum* merozoites.

Some *P*. *falciparum* strains mainly use ligands that bind to sialated receptors of the erythrocyte, other strains use ligands that bind to receptors independently of sialic acid. However, switching from sialic acid dependent to independent invasion is reversible and depends on the parasite ligand used [3,5]. Disruption of this binding through modification of the erythrocyte surface or by gene disruption has shown that the merozoites can use other invasion pathways [5,6]. This means that switching from sialic acid-dependent to sialic acid-independent invasion is possible but the genetic bases of this process are unknown [7,8]. One of the most essential *P. falciparum* merozoites ligands is the (EBA-175), which utilizes the sialated erythrocytes receptor, GPA, the major glycophorin found on human erythrocytes, in order to invade the red blood cell [1,9,10]. This antigen is located in the microneme organelles at the apical end of merozoites and belongs to a family of parasite adhesion molecules, the Duffy- Binding-like erythrocyte-binding proteins (DBL-EBP) [11]. EBA-175 consists of 7 regions, in region II at the N terminus there are two cystine rich segments (F1, F2) responsible for binding to the GPA [9,11]. It is well established that the gene that encodes the EBA-175 has a highly divergent dimorphic segments of sequences in region III, the first one detected in the FCR3 strains of *P. falciparum* "referred to as the F loop" and the second one in CAMP strains "referred to as the C loop" [12,13]. F and C segments which are inserted at different positions in the coding sequence of exon 1 encode 141 and 114 amino-acid respectively. These two divergent segments are conserved in all *P. falciparum* examined up to date and since merozoites are haploid; each parasite has a C or F segment, but not both or neither [14]. In spite of the role of this dimorphism in host-parasite interaction is unclear, different studies have shown that the initial interaction of merozoite invasion involves binding of F or C segment to the GPA backbone after binding of region II "of EBA-175" to sialic acid residues of GPA [14,15]. Furthermore, it has been reported that the majority of *P. falciparum* merozoites use the erythrocytic GPA receptor in the invasion process [16]. Studies to determine distribution of F and C segments dimorphism among parasite populations from five different African countries including Sudan, showed that Sudan has the most divergent population with C allele frequency of 0.73 [17]. Another study among Ghanaian children showed that the C segment is not associated with severe malaria but confers a higher risk of fatal disease [18].

Therefore, this study was designed to study firstly, the distribution of the divergent dimorphic alleles of *P. falciparum* EBA-175 (F and C) in three different malaria endemic areas in Central and Eastern Sudan and secondly, the possible association of this dimorphism with the outcome of the disease.

## Methods

### Study area and subjects

This study is a cross-sectional carried out in November 2007 and December 2009. It was conducted in three regions; in the Eastern region (Um-Salala village) and the Central region of the Sudan (Wad Madani and Sennar). Um-Salala is a small village inhabited by a single tribe of ~1,200 people by 1997, and characterized by stable malaria transmission. Wad Madani and Sennar are two big cities located in central Sudan with a population size of 452,628 and 1.2 million respectively. Both are inhabited by multiple tribes and characterized by unstable malaria transmission [19].

The study was approved by the Ethical committee of the institute of endemic diseases, University of Khartoum. Informed consents were obtained from adults or parents of children before they were enrolled in the study. A questionnaire form includes information about the family and clinical picture for each participant was completed. Peripheral blood samples were collected from a total of 339 microscopically confirmed *P. falciparum* infected malaria patients from the three different malaria endemic areas; Um-Salala 57 samples, Sennar 166 samples and Wad Madani 116 samples. Depending on the World Health Organization (WHO) criteria of severe malaria, a report revised in the year 2000 [20] symptomatic severe malaria, symptomatic uncomplicated malaria, and asymptomatic malaria of all ages were involved. Two ml of whole blood samples were collected in heparinized tubes from all confirmed malaria cases with a positive blood film (BF). Another blood film and Immunochromatography test (ICT) (BinaxNOW® Malaria test) were performed according to the manufacturer instructions, not only to confirm the infection, but also to differentiate between different malaria plasmodium species, as *P. falciparum* is the only species under study. The BinaxNOW® Malaria ICT test targets the histidine-rich protein II (HRPII) antigen specific to *P. falciparum* and a pan-malarial antigen (PMA), common to all four malaria species capable of infecting humans - *P. falciparum, P. vivax, P. ovale, and P. malariae*.

### DNA extraction

Chelex extraction method was used for DNA extraction from whole blood spotted and dried on filter papers. Each filter paper punch was incubated overnight at 4°C in 1 mL of 0.5% saponin in phosphate-buffered saline (PBS). The punches were washed for 30 minutes in 1 mL PBS at 4°C, transferred into new tubes containing 25 μL of 20% Chelex-100 and 75 μL of distilled water, and vortexed for 30 seconds. The tubes were heated at 99°C for 15 minutes to elute DNA, vortexed, and centrifuged at 10,000 × *g* for 2 minutes. The supernatants, which contained the DNA, were carefully removed and transferred into new tubes [21].

### Nested PCR and product analysis

In order to confirm the infection with *P. falciparum,* nested PCR that was used as described previously [22] using 10 mM of each of the following universal oligonucleotide primers; inner PCR forward Primer (rPLU1): 5′tcaaagattaagccatgcaagtga3′, inner PCR reverse primer (rPLU5): 5′cctgttgttgccttaaacttc3′, outer PCR forward Primer (rFAL1): 5′ttaaactggtttgggaaaaccaaatatatt3′, and outer PCR reverse primer (rFAL2): 5′acacaatgaactcaatcatgactacccgtc3′. The samples that showed specific *P. falciparum* bands of 205 bp in 2% agarose were selected for a further nested PCR to genotype the EBA-175 dimorphism using specific primers described previously [23] as follow (GenBank database, accession number L7755): EBA1 forward (nucleotides 2336–2356) 5′caagaagcagttcctgaggaa3′ EBA2 reverse (nucleotides 3060–3083) 5′tctcaacattcatattaacaattc3′ for the first amplification (PCR); EBA3 forward (nucleotides 2351–2364) 5′gaggaaaacactgaaatagcacac3′ and EBA4 reverse (nucleotides 3042–3065) 5′caattcctccagactgttgaacat3′ for the second (nested) amplification. The following protocol was used for both the first and nested PCR; Hot start at 95°C for 5 minutes. Thirty cycles of denaturation at 95°C for 1 minute, primer annealing at 58°C for 1 minute, extension at 70°C for 2 minutes, and final extension at 72°C for 10 minutes. The volume used for each reaction is 25 μl. A master mix containing all reagents, except DNA, is prepared and aliquoted into the reaction tubes. The master mix for the first and nested PCR contains the same following reagents for one single test tube except for the set of the primers: 1.5 mM MgCl_2_, 2.5 μl of 10X PCR buffer, 10 mM dNTPs, 10 mM from each primer, 2 μl DNA, and 5 Unit Taq polymerase, 1 μl bovine serum albumin and 14.8 μl of dH_2_O. The second PCR end products were separated in agarose gel electrophoresis at 1.5% concentration and visualized using a gel documentation system (BioDoc-It Imaging System, Cambridge, UK) for the presence of 714 bp or 795 bp bands representing the C and F segments respectively. The presence of both bands indicates a co-infection with at least two different *P. falciparum* clones.

### Statistical analysis

Statistical analysis was carried out using the SPSS software version 9. F and C Alleles frequencies among populations were calculated, firstly collectively in all populations, secondly in each of the three different study areas separately. In order to determine the possible association between allele distributions and severity of the disease, comparison between genotypes and phenotypes were carried out using the Chi-squared test. The same test was used to detect the significance of the distribution of different alleles among the overall population. The t-test was used in order to compare between the median-values.

## Results

### Demographics

The total number of participants recruited in this study was 339 of which 57 from Um-Salala, 166 from Sennar and 116 from Wad Madani. Patients were with different malaria phenotype were included: 276 mild, 37 Asymptomatic and 35 severe. The group of severe malaria includes: 20 individuals less than five years of age with severe anemia, hyperpyrexia, convulsion and hyperparasitemia; the other 15 individuals were more than 10 years of age with hyperpyrexia, convulsion, anemia and hypoglycemia. The age of patients from all three study areas ranged from 1 year to 85 years, where 80% of the patients were less than 20 years old. The age average was 11.86 ± 11.02. Sex ratio was 1:1. The haemoglobin levels ranged from 4.4 g/dl– 18.0 g/dl with an average of 10.1 ± 1.8, and glucose levels ranged from 23–205 mg/dl, with an average of 105.6 ± 33.9 mg/dl.

### Distribution of F and C genotypes in the study areas

**Figure 1:**
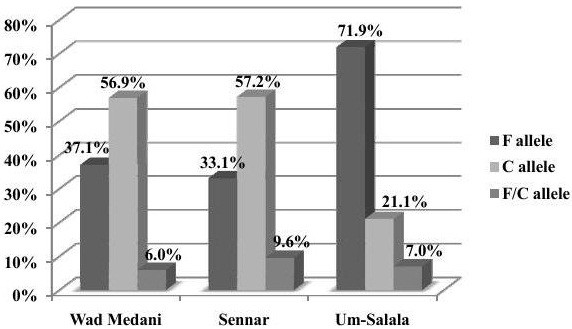
Distribution of F and C alleles in the three study areas.

A total of 339 *P. falciparum* positive blood samples from three different geographical areas have been examined for the dimorphic alleles F and C (Figure 1), they showed the following frequencies: 41.0% F, 51.0% C and 8.0% of the samples carried both F and C alleles.

In Sennar area, out of 166 samples, the prevalence of F, C, and mixed F/C genotypes were 55 (33.13%), 95 (57.23%), and 16 (9.64%) respectively. In Wad Madani, out of 116 samples, 43 (37.07%) were genotyped as F, 66 (56.90%) as C, and 7 (6.03%) as mixed F/C segments. The total number of samples from Um-Salala village were 57 samples out of which, 41(71.9%) showed the F allele, 12 (21.1%) were genotyped as C, and 4 (7.0%) carries a mixed alleles, F/C. The difference of the F and C alleles distribution among the different study areas collectively was significant (p-value = 0.00). No significant differences in the distribution of alleles in central Sudan-Sennar and Wad Madani (p-value = 0.4983).

The distribution of C and F alleles was assessed in four different age groups; less than 5 years, 5–10 years, 11–20 years and more than 20 years. No significant difference of C and F alleles frequencies within the different age groups (P-value = 0.28).

### Association of F and C genotypes with the phenotypes

**Figure 2:**
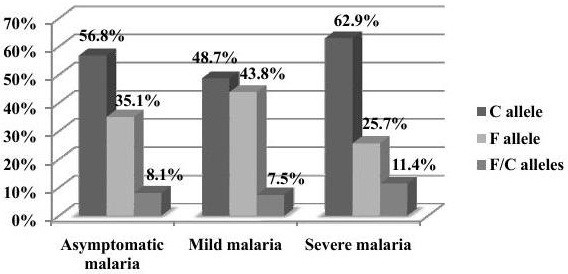
Association of the F and C alleles with malaria clinical manifestations.

Malaria phenotypes of all patients were categorized according to thecWHO criteria into three main groups: asymptomatic, mild, and severe malaria [20]. All malaria cases from Um-Salala were mild; the other two phenotypes were found in Sennar and Wad Madani (Figure 2). The Chi-squared test was used for all three groups collectively. There were no significant differences in the distribution of F and C alleles among the asymptomatic, mild and severe cases (p = 0.297). The genotypes distribution among Sennar mild cases were 33.13% F allele, 57.23% C allele and 8.64% carried both alleles, while there were no F among the five Sennar severe cases, but 3 of C and 2 of F/C alleles. Out of 116 malaria cases from Wad Madani, 37 were asymptomatic, 49 mild, and 30 severe cases. No association between genotypes and phenotypes was detected.

Eleven asymptomatic malaria patients from Wad Madani showed positive result for sickle cell test. The prevalence of F, C and mixed F/C segments among sicklers were 36.4%, 63.6%, and 0% respectively.

In Um-Salala village the 57 malaira patients were presented by mild malaria. The genotypes distribution was (72%) for the F allele, (21%) for the C allele and (7%) carried both alleles F/C.

### Association of F and C alleles with parasitemia

Using unpaired (independent) t- test, the parasitemia of 132 samples from central Sudan were related to F and C genotypes, the average parasitemia among C and F alleles carriers was 38317.9/μl, and 35485.7/μl respectively with no significant difference (P-value = 0.86).

## Discussion

Assessment of C and F distribution in 339 *P. falciparum* positive blood samples from three different geographical areas showed that both fragment types, C and F, had nearly the same distribution within the population of Wad Madani and Sennar, being 56.9% and 37.1%, respectively in Wad Madani and 57.23% and 33.13%, respectively in Sennar area. The percentage of the co-infection with both alleles C/F was 6% and 9.64% for Wad Madani and Sennar respectively. The parasites population in Wad Madani and Sennar, can be considered as a single gene pool because of their geographical proximity which may lead to an admixture. The two cities are close to each other so there is frequent population movement between these two big cities.

The distribution of the F allele in the third region (Um-Salala, Eastern Sudan), was markedly higher than that of the C allele. This could be due to location of Um-Salala village in remote area which is inaccessible during rainy season and could be described as semi isolated area. In addition to that this village is inhabited by one ethnic group of the Massalit tribe and in comparison with Sennar and Wad Madani their big cities subjected to population movement and this will lead to populations admixture and parasite diversity. Moreover there is only one single transmission peak symphonized with Anopheles arabiensis after the short rainy season in Um-Salala village [24]. This result disagrees with a previous study by Binks [17] that took place in Eastern Sudan (Daraweesh and Asar villages) and four other countries in Africa, where they reported that the distribution of the C allele in Eastern Sudan was markedly higher than that of F allele. However the high frequency of F allele in Um-Salala is similar to that obtained in the Lao PDR, Iran in Asia and Gabon and Burkina Faso in Africa [25-28]. A study took place in four malaria endemic areas in Burkina Faso among children under five years, showed that in both low and high malaria transmission seasons, F- allele was more prevalent than the C-allele. This implies that the distribution of F and C alleles is not affected by seasonal variation [28].

While the malaria pattern in Um-Salala is characterized by asymptomatic and mild forms, in Wad Madani the asymptomatic cases are relatively few and almost non for Sennar, where most of the cases were mild but some with severe symptoms.

In order to detect the effect of the dimorphic allele’s distribution on the out come of the disease, we compared the distribution to the phenotypes of the disease, which was categorized into three groups: Asymptomatic, mild, and severe malaria. There were no significant differences in the distribution of F and C among asymptomatic, mild, and severe clinical forms of malaria among whole populations from the three study areas collectively, p = 0.297. Our finding is similar to Heidari and his colleagues [27] as they concluded that there was no significant correlation between clinical outcomes and the EBA-175 fragment types in their study area in Iran. When eleven sickler cases infected with *P. falciparum* were genotyped for F and C fragments, we observed that the C allele was found to be more frequent than F allele, 7: 4 C to F respectively. This finding contradicts Cramer’s study [18] and suggests an uncoupling of the C allele and virulence, as the sickle cell trait shows strong resistance to severe and complicated malaria [29,30]. The Clinically protected human hosts, including sicklers, are known to get immunity faster than others due to modulated immune response [31,32]. Sicklers were found to have antiparasitic as well as anti inflammatory cytokine responses [33]. The higher frequency of the C allele among sicklers may suggest a survival advantage for these parasites in these clinically protected individuals since they never seek treatment. This survival advantage may render sicklers into safe reservoirs for the C allele parasites. If this is the case, sicklers would help in the wide distribution of the C allele parasites in the societies among whom the sickle gene exists. The overall results of the effect of sickle haemoglobin (HbS) trait on the differential selection of F and C alleles needs verification by increasing the sample size.

Furthermore, to find out the relationship between this dimorphism and severity of the disease, F and C distribution was correlated to the parasitemia level of subset of infected individuals. There was no significant difference in average of parasitemia in F and C allele’s carriers. This result is concurrence with Heidari *et al.*[26] who didn’t find significant correlation between parasitemia, gender, age of subjects and clinical outcome with the two fragments of EBA-175 gene.

## Conclusions

This study showed a significant differential distribution of F and C fragments in different geographical areas endemic with malaria. It could explain the different patterns of malaria in Um-Salala village and cities in central Sudan. More studies on larger populations are needed to understand the distributional pattern of EBA-175 alleles according to the clinical outcomes of the disease.

## Competing interests

The authors declare that they have no competing interests.

## Authors’ contributions

HSM conceived the study, substantial contributions to conception and design, field work, interpretation of data and drafting the manuscript. NHA participated in coordination of the study, recruitment of cases and helped in revising the manuscript. AAMA, involved in the collection of the samples, all field laboratory work, DNA extraction, genotyping and acquisition of data. DAH and WHO are involved in the recruitment of cases and sample collection. AAA contributes in all field laboratory work, sample collection and genotyping. BYN contributes in field station setting and sample collection. AZJ contributes in analysis of the data. MEI involved in coordination of the study and revising the manuscript. All authors read and approved the final manuscript.

## Pre-publication history

The pre-publication history for this paper can be accessed here:

http://www.biomedcentral.com/1471-2334/13/469/prepub

## References

[B1] Camus D Hadley TJ A *Plasmodium falciparum* antigen that binds to host erythrocytes and merozoites Science 1985 230 553 556 10.1126/science.3901257 3901257

[B2] Mitchell GH Hadley TJ McGinniss MH Klotz FW Miller LH Invasion of erythrocytes by *Plasmodium falciparum* malaria parasites: evidence for receptor heterogeneity and two receptors Blood 1986 67 1519 1521 3516259

[B3] Hadley TJ Klotz FW Pasvol G Haynes JD McGinniss MH *Falciparum* malaria parasites invade erythrocytes that lack glycophorin A and B (MkMk). Strain differences indicate receptor heterogeneity and two pathways for invasion J Clin Invest 1987 80 1190 1193 10.1172/JCI113178 3308959 PMC442364

[B4] Rayner JC Vargas-Serrato E Huber CS Galinski MR Barnwell JW A *Plasmodium falciparum* homologue of *Plasmodium vivax* reticulocyte binding protein (PvRBP1) defines a trypsin-resistant erythrocyte invasion pathway J Exp Med 2001 194 1571 1581 10.1084/jem.194.11.1571 11733572 PMC2193530

[B5] Duraisingh MT Maier AG Triglia T Cowman AF Erythrocyte-binding antigen 175 mediates invasion in *Plasmodium falciparum* utilizing sialic acid-dependent and -independent pathways Proc Natl Acad Sci USA 2003 100 4796 4801 10.1073/pnas.0730883100 12672957 PMC153635

[B6] Reed MB Caruana SR Batchelor AH Thompson JK Crabb BS Targeted disruption of an erythrocyte binding antigen in Plasmodium falciparum is associated with a switch toward a sialic acid-independent pathway of invasion Proc Natl Acad Sci USA 2000 97 7509 7514 10.1073/pnas.97.13.7509 10861015 PMC16576

[B7] Gaur D Mayer DC Miller LH Parasite ligand-host receptor interactions during invasion of erythrocytes by Plasmodium merozoites Int J Parasitol 2004 34 1413 1429 10.1016/j.ijpara.2004.10.010 15582519

[B8] Stubbs J Simpson KM Triglia T Plouffe D Tonkin CJ Molecular mechanism for switching of P. falciparum invasion pathways into human erythrocytes Science 2005 309 1384 1387 10.1126/science.1115257 16123303

[B9] Orlandi PA Sim BK Chulay JD Haynes JD Characterization of the 175-kilodalton erythrocyte binding antigen of Plasmodium falciparum Mol Biochem Parasitol 1990 40 285 294 10.1016/0166-6851(90)90050-V 2194125

[B10] Marchesi VT Tillack TW Jackson RL Segrest JP Scott RE Chemical characterization and surface orientation of the major glycoprotein of the human erythrocyte membrane Proc Natl Acad Sci USA 1972 69 1445 1449 10.1073/pnas.69.6.1445 4504356 PMC426722

[B11] Adams JH Sim BK Dolan SA Fang X Kaslow DC A family of erythrocyte binding proteins of malaria parasites Proc Natl Acad Sci USA 1992 89 7085 7089 10.1073/pnas.89.15.7085 1496004 PMC49650

[B12] Kain KC Lanar DE Determination of genetic variation within Plasmodium falciparum by using enzymatically amplified DNA from filter paper disks impregnated with whole blood J Clin Microbiol 1991 29 1171 1174 1864936 10.1128/jcm.29.6.1171-1174.1991 PMC269964

[B13] Sim BK Chitnis CE Wasniowska K Hadley TJ Miller LH Receptor and ligand domains for invasion of erythrocytes by Plasmodium falciparum Science 1994 264 1941 1944 10.1126/science.8009226 8009226

[B14] Ware LA Kain KC Lee Sim BK Haynes JD Baird JK Two alleles of the 175-kilodalton *Plasmodium falciparum* erythrocyte binding antigen Mol Biochem Parasitol 1993 60 105 109 10.1016/0166-6851(93)90033-T 8366884

[B15] Kain KC Orlandi PA Haynes JD Sim KL Lanar DE Evidence for two-stage binding by the 175-kD erythrocyte binding antigen of Plasmodium falciparum J Exp Med 1993 178 1497 1505 10.1084/jem.178.5.1497 8228803 PMC2191248

[B16] Baum J Pinder M Conway DJ Erythrocyte invasion phenotypes of *Plasmodium falciparum* in the Gambia Infect Immun 2003 71 1856 1863 10.1128/IAI.71.4.1856-1863.2003 12654801 PMC152018

[B17] Binks RH Baum J Oduola AM Arnot DE Babiker HA Population **genetic analysis of the *****Plasmodium falciparum *****erythrocyte binding antigen-175 (eba-175) gene** Mol Biochem Parasitol 2001 114 63 70 10.1016/S0166-6851(01)00240-7 11356514

[B18] Cramer JP Mockenhaupt FP Mohl I Dittrich S Dietz E Allelic dimorphism of the erythrocyte binding antigen-175 (eba-175) gene of Plasmodium falciparum and severe malaria: significant association of the C-segment with fatal outcome in Ghanaian children Malar J 2004 3 11 10.1186/1475-2875-3-11 15140262 PMC420250

[B19] Himeidan YE Elbashir MI Rayah E El A Adam I Epidemiology of malaria in New Halfa, an irrigated area in eastern Sudan East Mediterr Health J 2005 11 499 504 16602473

[B20] World Health Organization (WHO) Severe falciparum malaria Trans R Soc Trop Med Hyg 2000 94 1 11103309

[B21] Plowe CV Djimde A Bouare M Doumbo O Wellems TE Pyrimethamine and proguanil resistance-conferring mutations in Plasmodium falciparum dihydrofolatereductase: polymerase chain reaction methods for surveillance in Africa Am J Trop Med Hyg 1995 52 565 568 7611566 10.4269/ajtmh.1995.52.565

[B22] Snounou G Viriyakosol S Zhu XP Jarra W Pinheiro L High sensitivity of detection of human malaria parasites by the use of nested polymerase chain reaction Mol Biochem Parasitol 1993 61 315 320 10.1016/0166-6851(93)90077-B 8264734

[B23] Toure FS Mavoungou E Ndong JM Tshipamba P Deloron P Erythrocyte binding antigen (EBA-175) of *Plasmodium falciparum*: improved genotype determination by nested polymerase chain reaction Trop Med Int Health 2001 6 767 769 10.1046/j.1365-3156.2001.00789.x 11679124

[B24] Himeidan YE Elzaki MM Kweka EJ Ibrahim M Elhassan IM Pattern of malaria transmission along the Rahad River basin. Eastern Sudan Parasit Vectors 2011 4 109 10.1186/1756-3305-4-109 21679459 PMC3128851

[B25] Dittrich S Schwobel B Jordan S Vanisaveth V Rattanaxay P Distribution of the two forms of Plasmodium falciparum erythrocyte binding antigen-175 (eba-175) gene in Lao PDR Malar J 2003 2 23 10.1186/1475-2875-2-23 12901736 PMC169188

[B26] Toure FS Bisseye C Mavoungou E Imbalanced distribution of *Plasmodium falciparum* EBA-175 genotypes related to clinical status in children from Bakoumba, Gabon Clin Med Res 2006 4 7 11 10.3121/cmr.4.1.7 16595788 PMC1435655

[B27] Heidari A Keshavarz H Dittrich S Jelinek T Allelic Dimorphism of the *P. falciparum* Erythrocyte Binding Antigen 175 (EBA-175) Gene in the Southeast of Iran Iranian J Parasitol 2009 4 17 22

[B28] Soulama I Bougouma EC Diarra A Nebie I Sirima SB Low-high season variation in Plasmodium falciparum erythrocyte binding antigen 175 (eba-175) allelic forms in malaria endemic area of Burkina Faso Trop Med Int Health 2010 15 51 59 19891760 10.1111/j.1365-3156.2009.02415.x PMC2858779

[B29] Allison AC Polymorphism and Natural Selection in Human Populations Cold Spring Harb Symp Quant Biol 1964 29 137 149 10.1101/SQB.1964.029.01.018 14278460

[B30] Aidoo M Terlouw DJ Kolczak MS McElroy PD TerKuile FO Protective effects of the sickle cell gene against malaria morbidity and mortality Lancet 2002 359 1311 1312 10.1016/S0140-6736(02)08273-9 11965279

[B31] Bayoumi RA The sickle-cell trait modifies the intensity and specificity of the immune response against P. faleiparum malaria and leads to acquired protective immunity Med Hypotheses 1987 22 287 298 10.1016/0306-9877(87)90193-9 3295496

[B32] Verra F Simpore J Warimwe GM Tetteh KK Howard T Osier FH Bancone G Avellino P Blot I Fegan G Bull PC Williams TN Conway DJ Marsh K Modiano D Haemoglobin C and S role in acquired immunity against Plasmodium falciparum malaria PLoS One 2007 3 2(10) 10.1371/journal.pone.0000978 PMC1991593 17912355

[B33] Hassan DA Marques C Santos-Gomes GM DoRosario VE Mohamed HS Differential expression of cytokine genes among sickle-cell-trait (HbAS) and normal (HbAA) children infected with *Plasmodium falciparum* Ann Trop Med Parasitol 2009 103 283 295 10.1179/136485909X435049 19508746

